# Metabolic mechanism of astaxanthin biosynthesis in *Xanthophyllomyces dendrorhous* in response to sodium citrate treatment

**DOI:** 10.1186/s40643-023-00650-7

**Published:** 2023-04-26

**Authors:** Xueshan Pan, Tonggang Li, Baobei Wang, Shuhua Qi, Dandan Yang, Zheng Huang, Renfei Gao, Jingyan Li, Xueping Ling, Yinghua Lu

**Affiliations:** 1 Department of Biochemistry and Molecular Biology, School of Laboratory Medicine, Bengbu Medical College, Bengbu, People’s Republic of China; 2 Department of Hygiene, School of Public Health, Bengbu Medical College, Bengbu, People’s Republic of China; 3 College of Oceanology and Food Science, Quanzhou Normal University, Quanzhou, People’s Republic of China; 4 Department of Chemical and Biochemical Engineering, College of Chemistry and Chemical Engineering, Xiamen University, Xiamen, 361005 People’s Republic of China; 5 The Key Lab for Synthetic Biotechnology of Xiamen City, Xiamen University, Xiamen, People’s Republic of China

**Keywords:** *Xanthophyllomyces dendrorhous*, Astaxanthin, Na-citrate, Metabolomics, Gene expression

## Abstract

Astaxanthin is an important ketocarotenoid widely used in industries. However, its application is limited because of its low yield. Sodium citrate (Na-citrate), one of the major carbon sources for microorganisms, can promote cell growth and product accumulation. The basidiomycetous red yeast *Xanthophyllomyces dendrorhous* was thus used to study the effect of Na-citrate on cell growth and astaxanthin synthesis. The highest biomass and astaxanthin yield (6.0 g/L and 22.5 mg/L) were obtained in shake-flask when 3 g/L Na-citrate was added at 24 h and were 1.8 and 2.0 times higher than those of the control group, respectively. Furthermore, metabolomics and real-time reverse transcription PCR (qRT-PCR) analysis were conducted to study the metabolic pathways of *X. dendrorhous* in response to Na-citrate. The qRT-PCR assay revealed that Na-citrate facilitated glucose consumption, promoted the metabolic flux from glycolysis, and regulated the tricarboxylic acid (TCA) cycle, providing more energy and substrates for the synthesis of astaxanthin. The gene analysis revealed that adding Na-citrate significantly upregulated the expression of six key genes (*ICL*, *HMGS*, *crtE*, *crtYB*, *crtI,* and *crtS*) involved in pathways related to astaxanthin biosynthesis. These results suggest that exogenous Na-citrate treatment is a potentially valuable strategy to stimulate astaxanthin production in *X. dendrorhous*.

**Supplementary Information:**

The online version contains supplementary material available at 10.1186/s40643-023-00650-7.

## Highlights

- Na-citrate treatment significantly increases biomass and astaxanthin yield in *X. dendrorhous*.
- Na-citrate treatment enhances the glycolysis pathway in *X. dendrorhous*.
- Na-citrate treatment downregulates TCA cycle-associated metabolites in *X. dendrorhous*.
- Na-citrate treatment improves the transcription levels of key genes associated with astaxanthin biosynthesis.
- Exogenous Na-citrate treatment is a potential regulatory model for astaxanthin biosynthesis in *X. dendrorhous*

**Supplementary Information:**

The online version contains supplementary material available at 10.1186/s40643-023-00650-7.

## Introduction

Astaxanthin (3,3'-dihydroxy-4,4'-dione-β,β-carotene) is an important orange-red carotenoid pigment with high commercial and biotechnological interests because of its strong antioxidant activity and wide applications in the food, nutraceutical, pharmaceutical, aquaculture, and cosmetic industries (Chatragadda & Dufosse 2021; Kanwugu et al. 2022; Ramesh et al. 2022). The global astaxanthin market has grown significantly in recent years because of its widespread use and its various biological activities (Gervasi et al. 2019; Nutakor et al. 2022; Ramesh et al. 2019). The production of natural astaxanthin using microorganisms, including microalgae and yeast, has attracted more attention because it is safe, has a shorter production cycle, is environmentally friendly, and has large-scale industrial production (Bauer & Minceva 2021; Harith et al. 2020; Yu et al. 2022).

The basidiomycetous yeast *Xanthophyllomyces dendrorhous* (formerly known as *Phaffia rhodozyma*) has been widely studied as one of the best natural resources for producing astaxanthin (Amado & Vazquez 2015; Harith et al. 2020). The most challenging problem associated with astaxanthin production in *X. dendrorhous* is the low productivity of astaxanthin for commercial production. Therefore, new strategies for regulating *X. dendrorhous* to accumulate astaxanthin efficiently need to be explored (Torres-Haro et al. 2021b). Chemical stimulants have recently been reported to be effective and economical in improving the accumulation of high-value bioproducts in microorganisms, such as vitamins, trace elements, fungal elicitors, sucrose, and ethylene (Nutakor et al. 2022; Schewe et al. 2017; Wang et al. 2019). Phytohormones play a crucial role in the biosynthesis of astaxanthin and are effective at low concentrations and important for the growth of *X. dendrorhous* (Nutakor et al. 2022; Pan et al. 2020). Furthermore, the exogenous addition of glycerol, mevalonate, glutamate, citrate, succinate, and other molecules as inductors may affect astaxanthin synthesis (Li et al. 2020; Wang et al. 2019).

Tricarboxylic acid (TCA) metabolic intermediates, a class of organic acids, are an indispensable carbon skeleton for the biosynthesis of carotenoids and lipids in microorganisms (Chen et al. 2009). Exogenous application of some specific intermediates in the TCA cycle significantly stimulates various biological processes, such as cell growth and metabolism of fatty acids, carotenoids, and carbohydrates in *Haematococcus pluvialis* and *P. rhodozyma* (Du et al. 2021; Flores-Cotera et al. 2001). Zhu et al. reported that the addition of ammonium ferric citrate improved chlorophyll synthesis by 22.5% to provide more electrochemical potential energy in the green stage and alleviated photosystem II damage to maintain a high level of effective quantum yield by enhancing carotenoid production in *H. pluvialis* (Zhu et al. 2021). Yu et al. reported that succinic acid could significantly enhance the growth of *H. pluvialis*, promoting astaxanthin accumulation but reducing chlorophyll, carbohydrate, and protein levels (Yu et al. 2021). Sodium citrate (Na-citrate), one of the major carbon sources for microorganisms, can promote cell growth and product accumulation. However, there are only a few studies on the effects of Na-citrate on the growth of *X. dendrorhous*. According to Flores-Cotera et al., citrate is a possible precursor of acetyl-CoA production to promote astaxanthin synthesis in *P. rhodozyma* (sexual state *X. dendrorhous*) (Flores-Cotera et al. 2001). This study thus investigated the effect of exogenous Na-citrate treatment on the growth and astaxanthin accumulation in *X. dendrorhous*.

Comparative metabolomics is used to gain insights into regulatory mechanisms (Alcalde & Fraser 2018), potentially providing new intuitions into intracellular metabolites and genes to obtain an integrated view of a response mechanism. In this study, the effect of Na-citrate on cell growth and astaxanthin accumulation in *X. dendrorhous* was investigated using gas chromatography–mass spectrometry (GC–MS) combined with real‑time reverse transcription PCR (qRT-PCR) to evaluate the metabolite and gene expression related to astaxanthin accumulation and the profile of regulatory characteristics. The study aimed to understand the astaxanthin biosynthesis pathway, establish a new strategy to improve astaxanthin production and promote the industrial development of natural astaxanthin by *X. dendrorhous*.

## Materials and methods

### Chemicals

Astaxanthin, ds were extracted from the cell pelletsβ-Carotene, and all chemicals used for GC–MS analyses were purchased from Sigma-Aldrich (St. Louis, USA). The trimethylamine was purchased from Fisher Scientific (Leicestershire, UK). All other chemicals were obtained from Sinopharm Chemical Reagent Co., Ltd. (Shanghai, China).

### Yeast strain and growth conditions

The yeast strain *X. dendrorhous* UV3-721 (CGMCC No.3045) was sourced from the Key Lab for Synthetic Biotechnology of Xiamen City at Xiamen University, China.

*X. dendrorhous* was preserved in 20% glycerol at -80 °C and was maintained in a Yeast Extract Peptone Dextrose (YPD) medium supplemented with 20 g/L glucose, 10 g/L yeast extract, and 20 g/L peptone. *X. dendrorhous* was first cultivated in 250-mL Erlenmeyer flasks containing 30 mL YPD medium, and the culture was incubated in a rotary shaker (SUKUN SKY-200B, Shanghai, China) at 22 °C and 200 rpm for 48 h. *X. dendrorhous* was then cultured in 250-mL Erlenmeyer flasks containing 30 mL YPD medium at 22 °C for 24 h. Subsequently, a 4.5 mL seed culture was transferred into a 250-mL Erlenmeyer flask containing 50 mL fermentation medium and incubated at 22 °C and 200 rpm for 120 h (Pan et al. 2017). The fermentation medium had a pH of 6.0 and contained 20 g/L glucose, 0.2 g/L yeast extract, 0.5 g/L (NH_4_)_2_SO_4_, 1.0 g/L KH_2_PO_4_, 0.1 g/L NaCl, 0.5 g/L MgSO_4_·7H_2_O, and 0.1 g/L CaCl_2_·2H_2_O.

The secondary seed culture was then transferred into a 5-L bioreactor (Winpact, Major Science, USA) with a working volume of 3 L for fermenter culture. The fermentation process was controlled at 22 °C, with no pH control, 800 rpm stirring speed, 2 vvm aeration, and dissolved oxygen (DO) maintained at more than 30% using the dissolved oxygen cascade.

Na-citrate treatment was done by adding 2 g/L Na-citrate to the medium at 6 time points (0, 12, 24, 36, 48, and 60 h). Different concentrations of Na-citrate (0, 1.0, 1.5, 2.0, 2.5, 3.0, 4.0, and 5.0 g/L) were added after optimizing the optimal addition time to 24 h to examine the optimum concentration*.* Cultures without Na-citrate addition were used as the controls. Samples from the control and Na-citrate groups were collected every 24 h for biomass, glucose concentration, carotenoids, astaxanthin, and metabolite analysis until the total fermentation time reached 120 h. Data were presented as means ± standard deviation of three independent replicate cultures.

### Determination of cell growth and glucose concentration

Biomass was determined using the dry cell weight (DCW, g/L) as previously described (Pan et al. 2020). The cell suspension was centrifuged at 8000×*g* and 8 °C for 10 min, followed by washing the cell pellets twice using distilled water and drying at 100 °C until they attained a constant weight. The supernatant obtained after centrifugation was used to measure the glucose concentrations on an SBA-40D biosensor (Shandong, China).

### Astaxanthin extraction and HPLC analysis

Total carotenoids were extracted from the cell pellets using dimethylsulfoxide (DMSO) as previously described (Pan et al. 2017). Astaxanthin was determined following the method described by Xie et al. (2013) with modifications. In brief, astaxanthin was analyzed on an Agilent 1200 series HPLC system equipped with a UV detector (Agilent Technologies, USA) and a YMC30 RP-30 column (4.6 mm × 250 mm × 5 μm) operated at 8 °C with a mobile phase of 1 mL/min. The eluents were: (A) 3% ddH_2_O in methanol containing 0.05 M ammonium acetate and (B) 100% tert-butyl methyl ether. All eluents contained 0.1% (w/v) butylated hydroxytoluene and 0.05% triethylamine. Gradient elution was carried out as previously described (Pan et al. 2020). The UV detection wavelength was adjusted to 478 nm.

### Preparation of intracellular metabolite samples and derivatization

Metabolome analysis was performed following the procedures described by Li et al. with minor modifications (Li et al. 2018). Briefly, 40 mL of the samples collected at different time points (cells from the control and Na-citrate treated groups were collected every 24 h, i.e., 48, 72, 96, and 120 h) were quickly harvested and immediately mixed with 60 mL of pre-chilled methanol (-40 °C) to quench the culture. The quenched culture was then centrifuged at 8000×*g* and 4 °C for 10 min to collect the quenched cells. The cell pellets were washed twice using physiological saline (0.9% of sodium chloride solution, pre-chilled at 4 °C) and stored at −80 °C awaiting use.

Metabolome extraction and derivatization were done by first grinding 0.5 g of cells into a fine powder using liquid nitrogen, followed by the addition of 0.5 mL cold methanol (pre-chilled at −40 °C) to the cell powder (about 0.1 g). The mixture was mixed thoroughly for 30 s, subjected to liquid nitrogen freeze and thawing, and then centrifuged at 8000×*g* and −4 °C for 10 min to collect the supernatant. The last step was repeated, and both supernatants were subsequently pooled together. The supernatant was mixed with 50 μL of internal standard (adonitol in water, 0.2 mg/mL) and then dried in a vacuum freeze dryer for 24 h. Sample derivatization was performed according to the two-stage technique (Yu et al. 2016a, b) with moderate modifications. Briefly, 60 μL of methoxyamine hydrochloride in pyridine (20 mg/mL) was added to the dried sample, and the mixture was incubated at 37 °C for 2 h. The sample was then silylated for 2 h at 37 °C by adding 60 μL of N-methyl-N-trimethylsilyl-trifluoroacetamide (MSTFA).

### GC–MS analysis

The metabolite samples were analyzed using a gas chromatography–mass spectrometry (GC–MS) (Agilent Technologies 7890–5975, Sacramento, USA) equipped with a HP-5 ms capillary column (30 m × 250 μm × 0.25 μm, Agilent, Folsom, USA). Helium was used as carrier gas at a constant flow of 1 mL/min. Samples (1 μL) were injected into the HP-5 ms capillary column coated with 5% phenyl and 95% methylpolysiloxane in split mode at a split ratio of 1. The temperature of the injection port was set at 280 °C, while the mass spectrometer was operated at an ion source and interface temperatures at 200 °C and 280 °C, respectively. The mass scan range was 50–600 m/z. The column temperature was maintained at 70 °C for a 2-min delay, increased to 180 °C at a rate of 7 °C/min, increased to 250 °C at a rate of 5 °C/min, and then to 300 °C at a rate of 25 °C/min, and held for 8 min. All tests were replicated six times, each with three biological replicates from separate yeast cultures and two technical replicates.

Peaks with a high match value (> 750) and similarity greater than 65% were identified as metabolites. The relative levels of the metabolites were determined based on the characteristic ions of the selected peaks. All detected peaks were identified using alignments of mass spectra from the library of the National Institute of Standards and Technology (NIST, Gaithersburg, MD). The identified metabolites were normalized using the internal standard and biomass of cells to acquire the relative abundance of the metabolites. Partial least squares-discriminant analysis (PLS-DA) was subsequently performed to determine the metabolites contributing to differences between the control group and the Na-citrate group using the SIMCA-P software (Version 11.0, Umetrics, Sweden) for multivariate statistical Analysis. PLS-DA was adopted because it is the commonly used method for classification purposes and biomarker selection in metabolomics studies. A metabolite with a variable influence on the projection value (VIP) higher than 1 indicated it significantly contributed to the separation of groups in the PLS-DA models. Hierarchical cluster analysis (HCA) of the metabolites was subsequently performed using a heat map and the Cluster software (HemI 1.0, Chinese Academy of Sciences, China).

### RNA isolation and qRT-PCR

Takara MiniBEST Universal RNA Extraction Kit (ZYMO, California, USA) was used to extract total RNA from 1 mL of yeast cells at 36, 60, and 84 h. First-strand complementary DNA (cDNA) synthesis was then performed using a High-Capacity cDNA Reverse Transcription Kit (Applied Biosystems, USA). The concentration of the obtained cDNA was measured using a Spectrophotometer (Quawell Nanodrop Q6000, USA) and then normalized to 100 ng/mL for qRT-PCR analysis. The qRT-PCR assays were performed on an Applied Biosystems real-time PCR System to detect the expression of the genes. The cycling conditions were set as follows: initial denaturation at 94 °C for 30 s, followed by 40 cycles of denaturation and primer annealing at 94 °C for 5 s and 60 °C for 30 s, and dissociation curve analysis. The relative quantification of the gene expression levels was normalized to that of the actin gene using the comparative 2^−ΔΔCT^ method (Livak & Schmittgen 2001). The qRT-PCR primers used in this study were designed using the Vector NTI® Express Designer and are outlined in Additional file 1: Table S1. All tests were replicated six times, each with three biological replicates from separate yeast cultures and two technical replicates.

### Analysis of the abundance of intracellular reactive oxygen species (ROS)

The one-step Fluorometric Intracellular ROS Kit (Sigma-Aldrich, MAK143-KT) was used to detect intracellular ROS. The specific step was performed as previously described (Pan et al. 2020).

### Statistical analysis

All the tests in this study had three biological replicates from separate yeast cultures. Two-tailed t-tests were performed using the software package Statistica 6.0, and data were presented as means ± standard deviation of three independent replicates. The significance threshold was set at *P* < 0.05.

## Results and discussion

### Effects of Na-citrate on cell growth, residual glucose, carotenoids, and astaxanthin titer in *X. dendrorhous*

In this study, 2 g/L Na-citrate was added to the medium at several time points (0, 12, 24, 36, 48, and 60 h), and the growth of *X. dendrorhous* was monitored at 96 and 120 h, which was the total fermentation time. Notably, Na-citrate increased biomass at 96 and 120 h compared to the control group. Astaxanthin titer increased to a maximum value at 120 h when Na-citrate was added at 24 h of cultivation (Additional file 1: Fig. S1). Na-citrate (0, 1.0, 1.5, 2.0, 2.5, 3.0, 4.0, and 5.0 g/L) was added to the medium at 24 h after inoculation to explore the influence of Na-citrate on *X. dendrorhous*. The addition of Na-citrate had a significant effect on cell growth and increased the production of carotenoids and astaxanthin (Additional file 1: Fig. S2). Notably, the astaxanthin titer increased to 24.5 mg/L after adding 3 g/L Na-citrate at 24 h of cultivation, which was 85.2% higher than that of the control group at 120 h.

**Fig. 1:**
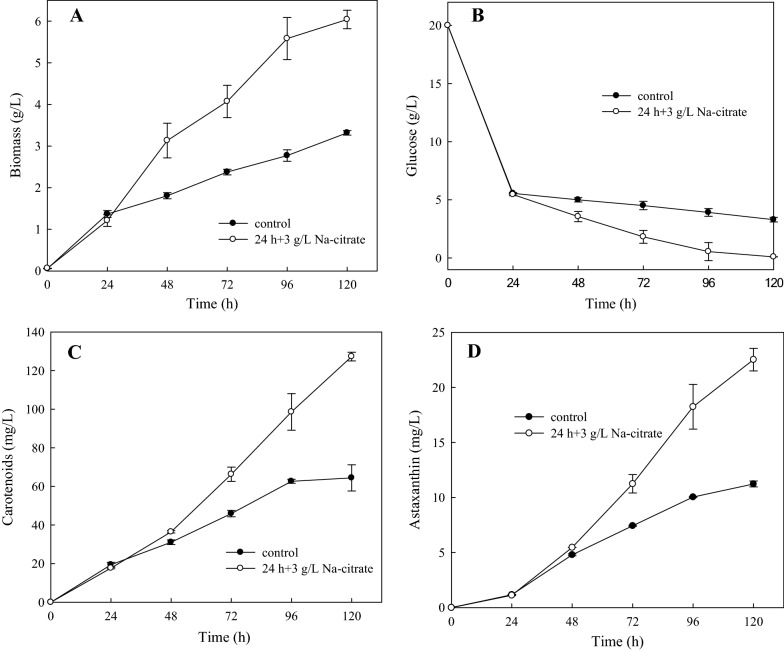
Time-course profiles of *X. dendrorhous* cultures in the Na-citrate and control groups. Control group: solid circles, Na-citrate group: hollow circles. **A** Biomass (g/L); **B** glucose (g/L); **C** carotenoids (mg/L); **D** astaxanthin (mg/L). The cells were grown in a 250-mL Erlenmeyer flask containing 50 mL fermentation medium, with the temperature maintained at 22 °C and the stirring speed at 200 rpm. Values are means ± standard deviation of three independent experiments

The time course of cell growth, residual glucose, carotenoids, and astaxanthin titer under both cultures was monitored after the addition of 3 g/L Na-citrate at 24 h to investigate the effects of Na-citrate on cell growth and astaxanthin biosynthesis during the entire fermentation process. Samples from the control and Na-citrate groups were collected every 24 h until the fermentation time reached 120 h. Na-citrate treatment achieved higher biomass, carotenoids, and astaxanthin titer compared to the control group (Fig. 1**)**. A rapid increase in biomass was observed in both cultures after 24 h, but the increase rate under Na-citrate treatment was higher than in the control group. The peak biomass after Na-citrate treatment was 6.0 g/L at 120 h, which was 81.93% higher than that in the control group **(**Fig. 1A**)**.

In addition, glucose was consumed quickly during the first 24 h after inoculation in both cultures at a consumption rate of 0.62 g/L/h. The consumption rate of glucose was analyzed every 24 h after adding Na-citrate for 24 h. *X. dendrorhous* cells utilized glucose quickly under Na-citrate conditions after 24 h, suggesting that the addition of Na-citrate promoted glucose utilization when cells entered the logarithmic growth phase, thus benefiting the cells to maintain a faster growth rate (Fig. 1B). The addition of Na-citrate increased carotenoids and astaxanthin accumulation after the increase of biomass and showed a twofold increase in contrast to the control group (Fig. 1C and D).

The regulation of Na-citrate to astaxanthin biosynthesis originates from increased cell growth. Na-citrate is a kind of carbon source stimulating cell growth and development (An., 2001; Du et al. 2021). In addition, Na-citrate is an inexpensive chemical that is metabolized by aerobic microorganisms to increase intracellular ATP levels, thereby enhancing the ability of cells to resist acid-stressed environments (Sánchez et al. 2008; Zhou et al. 2011). It can regulate the pH value of liquids during fermentation, thus aiding cell growth and astaxanthin production (Flores-Cotera et al. 2001).

**Fig. 2:**
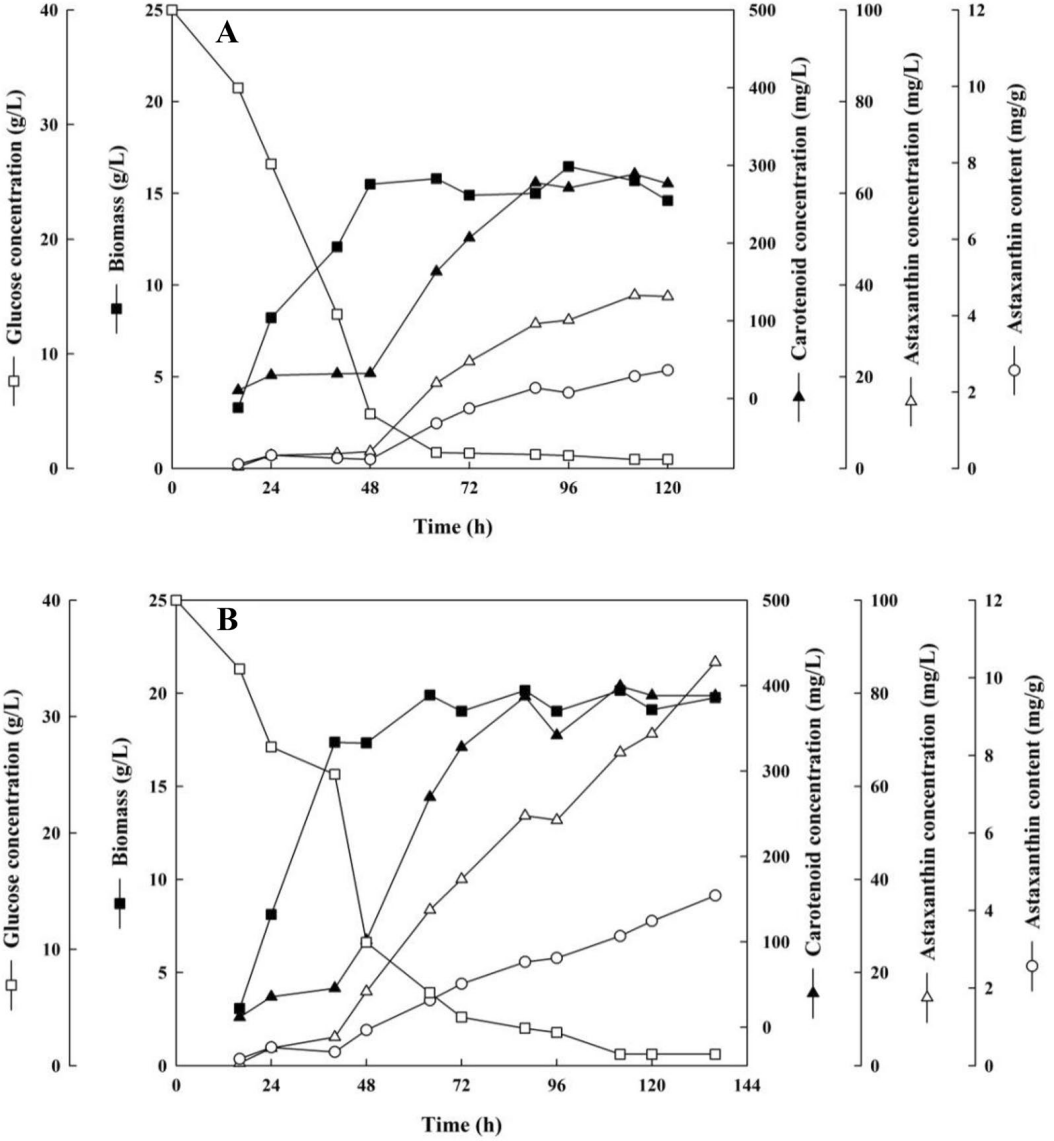
Profiles of cell growth, glucose consumption, and astaxanthin production of *X. dendrorhous* in the 5-L bioreactor. **A** Control group; **B** Na-citrate group. During the fermentation process, the temperature was maintained at 22 °C, the ventilation rate was 2 vvm, and the stirring speed was amplified step by step (0–24 h, 200 rpm; 24–48 h, 300 rpm; 48–72 h, 400 rpm; 72–120 h, 500 rpm), and the dissolved oxygen (DO) was maintained at more than 30% by the DO cascade. Glucose (open square), biomass (filled square), carotenoid titer (filled triangle), astaxanthin titer (open triangle), and astaxanthin content (open circle). The data shown are the average of two replicates (± standard deviation)

Herein, the batch fermentation in a 5-L fermenter was processed in two groups to mimic industrial production. For the control group (Fig. 2A), the cells entered the exponential growth phase after inoculation and reached the stationary phase at 48 h. Glucose concentration decreased before 48 h, which potentially contributed to the fast cell growth (logarithmic phase) during this period. Glucose consumption was slow after 48 h. At the same time, the carotenoid accumulation in *X. dendrorhous* increased significantly, and the titer achieved 294 mg/L at 120 h. Besides, astaxanthin production was closely parallel with the carotenoid accumulation and reached 44.2 mg/L at 120 h. Compared to the control batch fermentation, there was still a slight increase in biomass after rapid growth at 48 h and reached a maximum of 20.2 g/L after the addition of Na-citrate. This result was also observed from the change of residual glucose, in which the glucose consumption rate of the Na-citrate group was higher than that of the control group between 48 and 72 h (Fig. 2B). Furthermore, the carotenoids and astaxanthin started to accumulate at 8 h because of the rapid growth of cells. The productivity of carotenoids between 40 and 48 h was 0.38 mg/L/h, which was significantly higher than that of the control group. The biomass, carotenoid, and astaxanthin titer were 1.26-, 1.32-, and 2.01-fold higher, respectively, than the corresponding values in the control group. The astaxanthin content increased by 48.1% at 120 h after the addition of Na-citrate. These results indicated that Na-citrate treatment could promote the growth of cells and facilitate astaxanthin synthesis. *X. dendrorhous* cultivation using Na-citrate in the fermenter is a promising strategy for astaxanthin accumulation. Future studies should focus on optimizing fermentation parameters, such as temperature, rotation speed, pH, and DO levels, to enhance astaxanthin production and increase the benefits of enterprises. Moreover, these strategies can also be studied in combination with other methods, such as analysis of kinetic parameters of fermentation processes and performing kinetic modeling to guide actual industrial production to increase astaxanthin accumulation.

Table 1 compares the fermentation results of this study and other studies, excluding the results of genetic modification fermentation. The astaxanthin content obtained in this study was higher than that of previously published reports, except the results by de la Fuente et al. (2010), who obtained 4.77 mg/g by culturing *P. rhodozyma* VKPM 2476 in a 10-L fermenter. The increased astaxanthin content in a unit cell indicated that Na-citrate could regulate cell growth of *X. dendrorhous* by inducing crucial metabolic pathways associated with astaxanthin biosynthesis. Metabolome analysis was thus carried out to determine the changes caused by Na-citrate at the metabolite level in *X. dendrorhous*.

**Table 1:** Comparison of biomass, astaxanthin titer, and astaxanthin content in *X. dendrorhous* with those reported in the literature

Yeast	Cultivation method	Biomass (g/L)	Astaxanthin (mg/L)	Astaxanthin content (mg/g)	Refs.
ATCC 74219	Shake-flask	6.3	22.5	3.56	(Amado & Vazquez 2015)
NBRC	Shake-flask	17.0 (OD_600_)	2.5	0.39	(Hara et al. 2014)
NBRC 10129	Shake-flask	5.5	1.7	0.30	(Yamamoto et al. 2016)
UV3-721	Shake-flask	3.3	15.6	4.67	(Pan et al. 2020)
UV3-721	Shake-flask	3.1	3.5	1.14	(Wang et al. 2019)
PR106	Shake-flask	–	–	2.53	(Zhang et al. 2019)
JH1	Shake-flask (1.4 L)	37.1	45.6	1.22	(Kim et al. 2005)
25-2	Bioreactor (2 L)	8.8	2.3 (carotenoid)	0.26 (carotenoid content)	(Torres-Haro et al. 2021a)
25-2	Bioreactor (110 L)	9.0	1.9 (carotenoid)	0.22 (carotenoid content)	(Torres-Haro et al. 2021a)
AXJ-20	Bioreactor (20 L)	22.1	85	3.90	(Schewe et al. 2017)
DSMZ 5626	Bioreactor (2 L)	16.4	3.6	0.22	(Harith et al. 2020)
ZJUT46	Bioreactor (20 L)	15.7	27.1	1.74	(Hu et al. 2006)
ZJUT46	Bioreactor (1500 L)	17.4	39.5	2.20	(Hu et al. 2007)
2A2N	Bioreactor (100 L)	36.0	40.0	1.10	(An., 2001)
ZJUT003	Bioreactor (35 L)	21.3	58.8	2.77	(Zheng et al. 2006)
E5042	Bioreactor (35 L)	30.7	77	2.51	(Liu et al. 2008)
VKPM 2476	Bioreactor (10 L)	88	420	4.77	(de la Fuente et al. 2010)
AS 2.1557	Bioreactor (10 L)	17.3	14.3	0.80	(Wang & Yu 2009)
ENM5	Bioreactor (1 L)	29.2	26.8	0.92	(Liu et al. 2008)
**UV3-721**	**Bioreactor (3 L)**	**20.2**	**87**	**4.38**	**This study**

### Metabolic changes of *X. dendrorhous* cultured with and without Na-citrate

**Fig. 3:**
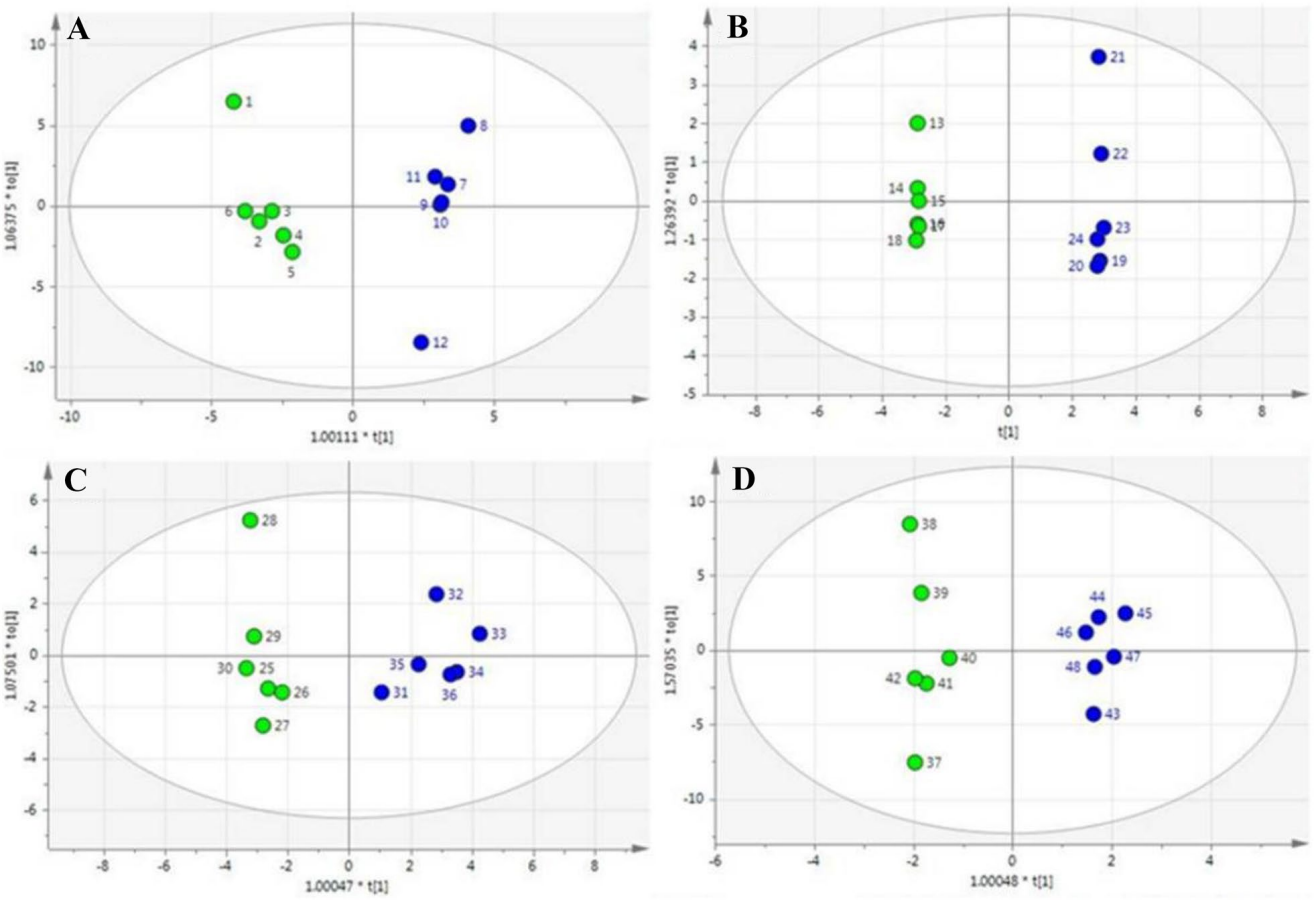
PLS-DA derived plots for pairwise comparisons between the Na-citrate and control groups at various time points: **A** 48 h, **B** 72 h, **C** 96 h, and **D** 120 h. Green circle: control groups. Blue circle: Na-citrate-treatment groups

**Fig. 4:**
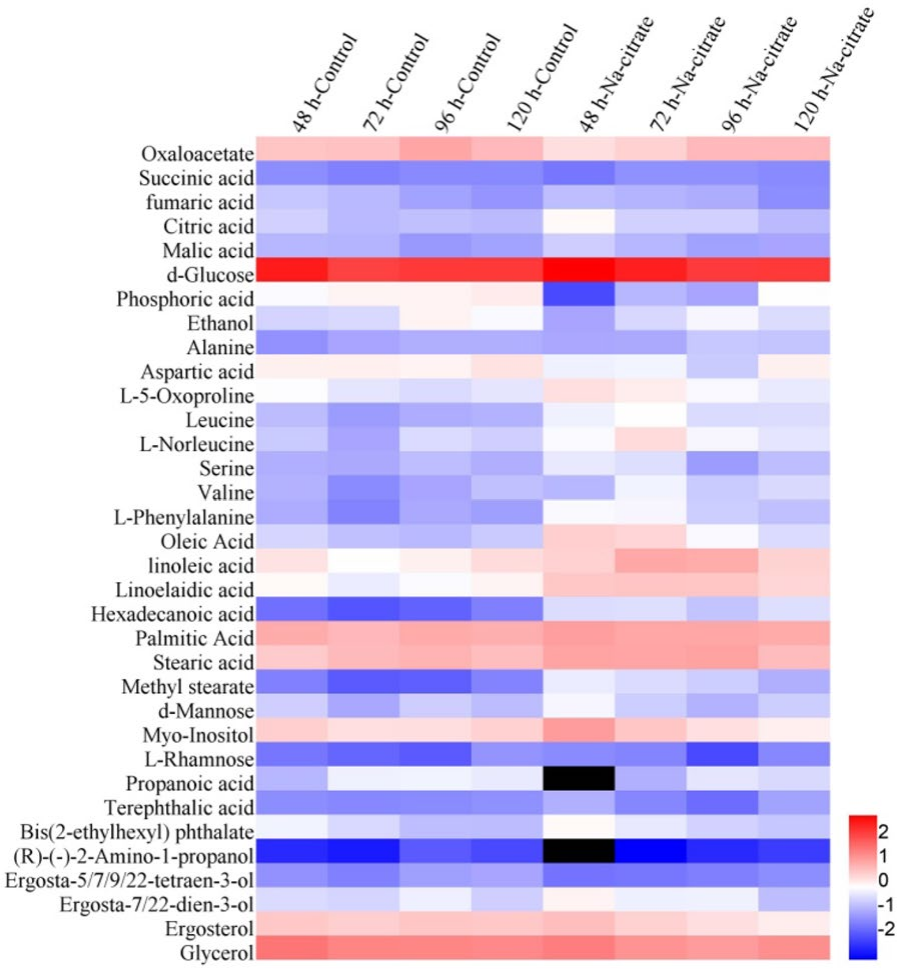
Hierarchical cluster analysis (HCA) for the identified metabolites. All data are expressed as the means of six replicates. The response ratio for each metabolite is normalized to log10

Metabolic profiles of *X. dendrorhous* were analyzed using a partial least squares-discriminant analysis (PLS-DA) score plot (Fig. 3). Analysis of the score plots revealed the metabolomic profiles of the control and the Na-citrate treatment groups were separated at all four-time points. A total of 34 chemically classified metabolites with a very important variable of projection (VIP) value greater than 1 and *P* values less than 0.05, including organic acids, amino acids, carbohydrates, fatty acids, and ergosterol, were analyzed (Tables 2 and 3). Most metabolites were involved in the TCA cycle, carbohydrate metabolism, fatty acid synthesis, and amino acid metabolism. Figure 4 is a heat map showing their functional classification.

**Table 2:** The metabolites responsible for responding to Na-citrate in *X. dendrorhous*

Metabolite	48 h		72 h		96 h		120 h	
	Control	Na-citrate	Control	Na-citrate	Control	Na-citrate	Control	Na-citrate
Oxaloacetate	4.65 ± 0.62	2.24 ± 0.31*	4.94 ± 0.41	3.22 ± 0.17**	10.65 ± 0.30	6.06 ± 0.93	6.33 ± 0.14	6.26 ± 0.76
Succinic acid	0.04 ± 0.01	0.02 ± 0.00**	0.03 ± 0.01	0.05 ± 0.01**	0.04 ± 0.00	0.04 ± 0.01	0.04 ± 0.01	0.04 ± 0.01
Fumaric acid	0.20 ± 0.04	0.16 ± 0.02*	0.14 ± 0.01	0.20 ± 0.03**	0.08 ± 0.02	0.10 ± 0.01*	0.05 ± 0.02	0.04 ± 0.01
Citric acid	0.27 ± 0.01	0.88 ± 0.03*	0.14 ± 0.01	0.39 ± 0.01**	0.17 ± 0.02	0.21 ± 0.02 *	0.15 ± 0.01	0.35 ± 0.02
Malic acid	0.13 ± 0.02	0.22 ± 0.01**	0.13 ± 0.03	0.15 ± 0.03	0.06 ± 0.04	0.04 ± 0.03*	0.08 ± 0.01	0.05 ± 0.01
d-Glucose	412 ± 28	853 ± 54*	152 ± 35	383 ± 53**	186 ± 21	183 ± 24	191 ± 54	192 ± 26
Phosphoric acid	0.83 ± 0.51	0.01 ± 0.00*	1.29 ± 0.65	0.14 ± 0.10**	1.33 ± 0.81	0.09 ± 0.01**	1.59 ± 0.23	0.98 ± 0.15
Ethanol	0.29 ± 0.03	0.08 ± 0.01	0.34 ± 0.07	0.33 ± 0.05	1.34 ± 0.51	0.73 ± 0.29*	0.81 ± 0.31	0.37 ± 0.01
Alanine	0.05 ± 0.01	0.09 ± 0.02	0.08 ± 0.02	0.10 ± 0.05	0.11 ± 0.06	0.21 ± 0.01*	0.11 ± 0.01	0.19 ± 0.03
Aspartic acid	1.42 ± 0.18	0.63 ± 0.01*	1.49 ± 0.72	0.68 ± 0.21*	1.30 ± 0.22	0.23 ± 0.10*	2.01 ± 0.22	1.43 ± 0.31
L-5-Oxoproline	0.87 ± 0.23	2.19 ± 0.63**	0.47 ± 0.04	1.54 ± 0.59*	0.34 ± 0.02	0.77 ± 0.18*	0.47 ± 0.21	0.53 ± 0.23
Leucine	0.16 ± 0.09	0.64 ± 0.23**	0.07 ± 0.02	0.98 ± 0.92**	0.11 ± 0.47	0.35 ± 0.06**	0.12 ± 0.07	0.37 ± 0.29
L-Norleucine	0.23 ± 0.17	0.83 ± 0.31	0.08 ± 0.04	2.36 ± 1.26**	0.36 ± 0.01	0.75 ± 0.12	0.26 ± 0.18	0.46 ± 0.44
Serine	0.11 ± 0.04	0.49 ± 0.22**	0.09 ± 0.03	0.38 ± 0.35	0.16 ± 0.23	0.07 ± 0.02	0.11 ± 0.07	0.17 ± 0.21
Valine	0.12 ± 0.06	0.13 ± 0.16	0.04 ± 0.01	0.67 ± 0.06**	0.08 ± 0.05	0.22 ± 0.05**	0.18 ± 0.09	0.33 ± 0.16
L-Phenylalanine	0.10 ± 0.04	0.77 ± 0.27**	0.03 ± 0.01	0.74 ± 0.42**	0.09 ± 0.14	0.24 ± 0.03	0.07 ± 0.04	0.17 ± 0.11
Oleic acid	0.32 ± 0.08	3.45 ± 1.67**	0.17 ± 0.04	2.91 ± 0.09**	0.14 ± 0.06	0.77 ± 0.1**	0.22 ± 0.07	0.36 ± 0.25
linoleic acid	2.01 ± 0.93	3.32 ± 0.73	0.98 ± 0.17	10.06 ± 0.84*	1.39 ± 0.95	8.51 ± 1.9*	2.36 ± 0.67	3.04 ± 0.75
Linoelaidic acid	1.11 ± 0.55	4.23 ± 2.02**	0.56 ± 0.09	4.47 ± 0.28**	0.81 ± 0.55	4.34 ± 0.89**	1.33 ± 0.37	2.95 ± 1.66
Hexadecanoic acid	0.02 ± 0.01	0.36 ± 0.02**	0.01 ± 0.00	0.38 ± 0.06**	0.01 ± 0.00	0.19 ± 0.07**	0.03 ± 0.00	0.38 ± 0.03**
Palmitic acid	8.7 ± 1.6	13.2 ± 1.6*	6.4 ± 1.8	10.1 ± 1.2**	8.9 ± 2.9	10.3 ± 1.5	8.1 ± 1.5	9.1 ± 1.1
Stearic acid	3.86 ± 0.84	11.13 ± 2.27**	5.86 ± 0.48	10.34 ± 1.17**	7.32 ± 1.73	11.62 ± 1.75**	5.40 ± 0.67	5.67 ± 1.38
Methyl stearate	0.03 ± 0.01	0.56 ± 0.18**	0.01 ± 0.00	0.35 ± 0.06**	0.01 ± 0.00	0.24 ± 0.08**	0.03 ± 0.00	0.11 ± 0.09
d-Mannose	0.26 ± 0.09	0.73 ± 0.43*	0.09 ± 0.02	0.24 ± 0.09**	0.25 ± 0.29	0.12 ± 0.04	0.15 ± 0.09	0.24 ± 0.10
Myo-Inositol	3.39 ± 0.48	13.31 ± 1.84**	2.16 ± 0.21	4.46 ± 0.35*	2.30 ± 0.77	2.18 ± 0.15	3.26 ± 0.69	1.43 ± 0.09
L-Rhamnose	0.03 ± 0.01	0.04 ± 0.01**	0.02 ± 0.011	0.04 ± 0.02	0.01 ± 0.03	0.01 ± 0.00	0.05 ± 0.033	0.04 ± 0.02**
Propanoic acid	0.13 ± 0.06	0.00 ± 0.00*	0.63 ± 0.06	0.11 ± 0.01	0.66 ± 0.04	0.46 ± 0.10**	0.53 ± 0.06	0.34 ± 0.04*
Terephthalic acid	0.05 ± 0.01	0.12 ± 0.01**	0.04 ± 0.01	0.04 ± 0.01	0.04 ± 0.00	0.02 ± 0.00	0.05 ± 0.00	0.08 ± 0.00**
Bis(2-ethylhexyl) phthalate	0.67 ± 0.12	1.09 ± 0.19**	0.33 ± 0.08	0.50 ± 0.07**	0.17 ± 0.07	0.28 ± 0.07	0.16 ± 0.14	0.21 ± 0.02
(R)-(-)-2-Amino-1-propanol	0.00 ± 0.00	0.00 ± 0.00*	0.00 ± 0.00	0.00 ± 0.00*	0.01 ± 0.00	0.00 ± 0.00*	0.01 ± 0.00	0.01 ± 0.00
Ergosta-5,7,9,22-tetraen-3-ol	0.05 ± 0.05	0.02 ± 0.01	0.03 ± 0.01	0.02 ± 0.01**	0.07 ± 0.06	0.03 ± 0.02	0.08 ± 0.03	0.04 ± 0.02*
Ergosta-7,22-dien-3-ol	0.35 ± 0.06	1.31 ± 0.16*	0.31 ± 0.10	0.61 ± 0.14	0.61 ± 0.21	0.64 ± 0.11	0.26 ± 0.09	0.17 ± 0.02
Ergosterol	4.05 ± 0.27	5.40 ± 0.14	3.47 ± 0.64	3.32 ± 0.67	4.45 ± 0.40	2.30 ± 0.43*	4.13 ± 0.19	1.52 ± 0.25
Glycerol	37.5 ± 11.6	30.6 ± 14.0	24.8 ± 2.1	17.3 ± 3.7**	23.9 ± 6.9	13.5 ± 2.5**	23.4 ± 2.2	18.9 ± 10.3

The data represent the contents of metabolites (mg/g DCW) and are presented as the means ± standard deviation. Statistical significance was estimated by *t*-test. *p < 0.05 compared with the control; **p < 0.01 compared with the control

**Table 3:** Relative changes in the cell metabolite abundance when cultured with Na-citrate versus control

Metabolites	Fold change Na-citrate/control*			
	48 h	72 h	96 h	120 h
TCA cycle				
Oxaloacetate	0.48	0.65	0.57	0.99
Succinic acid	0.55	1.5	0.98	1
Fumaric acid	0.81	1.45	1.32	0.8
Citric acid	3.25	2.72	1.22	2.38
Malic acid	1.66	1.18	0.71	0.59
Glycolysis pathway				
d-Glucose	2.07	2.52	0.98	1.01
Phosphoric acid	0.01	0.11	0.06	0.62
Ethanol	0.28	0.96	0.55	0.46
Amino acids				
Alanine	1.84	1.18	1.93	1.76
Aspartic acid	0.44	0.46	0.17	0.71
L-5-Oxoproline	2.53	3.29	2.23	1.13
Leucine	4.06	14.9	3.36	3.13
L-Norleucine	3.6	28.1	2.06	1.75
Serine	4.51	4	0.41	1.56
Valine	1.09	16	2.69	1.9
L-Phenylalanine	7.48	22.4	2.58	2.4
Fatty acids				
Oleic acid	10.8	16.9	5.42	1.61
linoleic acid	1.66	10.27	6.14	1.29
Linoelaidic acid	3.82	7.98	5.34	2.21
Hexadecanoic acid	18.2	38.2	13.4	12.7
Palmitic acid	1.52	1.59	1.16	1.12
Stearic acid	2.89	1.77	1.59	1.05
Methyl stearate	17.3	31.8	18.4	3.24
Sugars				
d-Mannose	2.81	2.7	0.49	1.58
Myo-Inositol	3.93	2.07	0.95	0.44
L-Rhamnose	0.73	1.37	0.74	0.67
Organic acids				
Propanoic acid	0	0.18	0.7	0.64
Terephthalic acid	2.58	0.95	0.46	1.69
Bis(2-ethylhexyl) phthalate	1.64	1.53	1.67	1.34
Others				
R)-(-)-2-Amino-1-propanol	0	0.5	0.27	0.71
Ergosta-5,7,9,22-tetraen-3-ol	0.43	0.75	0.43	0.51
Ergosta-7,22-dien-3-ol	3.72	1.99	1.04	0.64
Ergosterol	1.33	0.96	0.52	0.37
Glycerol	0.82	0.7	0.57	0.81

^*^Mean fold changes in Na-citrate compared with control. Statistical significance was estimated by *t*-test (*p* < 0.01), which is shown as bold values

### Effect of Na-citrate on the glycolysis pathway in *X. dendrorhous*

There was a clear metabolite tendency for the intracellular glycolysis pathway. In Na-citrate cultures, the glycolysis pathway was significantly upregulated **(**Tables 2 and 3**)** because of the rapid absorption of glucose from the medium induced by Na-citrate addition. The concentrations of intracellular glucose in the cells were increased by 107.6% (*P* < 0.05) at 48 h and 151.6% (*P* < 0.05) at 72 h (Table 2). The intracellular glucose in the Na-citrate group was at a similar level to that of the control group after 96 h despite the Na-citrate group consuming more glucose, suggesting that the addition of Na-citrate led to an accelerated rate of glucose uptake for cell growth at the early and middle stages of the fermentation. In contrast, the absorbed glucose at the later stage was utilized for its metabolic activities.

In addition, the content of ethanol, a product of yeast anaerobic fermentation, in the Na-citrate group was significantly lower than that in the control group (Tables 2 and 3). This finding suggested that a large part of the pyruvate generated by the glycolysis pathway was directly converted into acetyl-CoA rather than entering the anaerobic fermentation to accumulate ethanol. An increase of acetyl-CoA may cause the carbon metabolism to flow more to the fatty acid synthesis pathway and astaxanthin synthesis pathway because it is a key substrate of various cellular processes. Notably, the addition of Na-citrate resulted in a significant decrease in the intracellular phosphate concentration (Table 2). Flores-Cotera et al. (2001) reported that phosphate-limiting conditions affect RNA synthesis, which influences the DNA replication rate and protein biosynthesis, causing key substrates and coenzymes (acetyl-CoA, ATP, and NADPH) to be available for other metabolisms, including the TCA cycle, fatty acid synthesis, and carotenoid synthesis. This report is consistent with the results of our study.

### Effect of Na-citrate on the TCA cycle in *X. dendrorhous*

During the cultivation period, most intermediates of the TCA cycle were significantly decreased upon Na-citrate treatment besides citric acid and malic acid (Tables 2 and 3), indicating that Na-citrate reduced the metabolic flux toward the TCA cycle. Na-citrate treatment enhanced the concentration of citric acid, inhibiting the catalytic activity of citrate synthase from synthesizing citric acid and weakening the TCA cycle. In the oleaginous yeasts and fungi, low TCA activity induces citric acid accumulation in the mitochondria, which is then transported into the cytoplasm. In the cytoplasm, citric acid is degraded into acetyl-CoA and oxaloacetate under ATP citrate lyase (ACL) catalysis, thus promoting the production of fatty acids and carotenoids in *X. dendrorhous* (Venkateshwaran et al. 2015). Citrate is thus considered the precursor of acetyl-CoA for fatty acid and astaxanthin synthesis (Chavez-Cabrera et al. 2010; Ratledge & Wynn 2002). In the cytoplasm, oxaloacetate can be converted into malic acid by malate dehydrogenase (NADP^+^) and used for pyruvate and NADPH production (Chen et al. 2009). Pyruvate can be converted into acetyl-CoA through the pyruvate dehydrogenase complex, while the NADPH can be used for fatty acid synthesis. Exogenous Na-citrate may provide more acetyl-CoA by cleaving citrate to produce acetyl-CoA and reduce the consumption of acetyl-CoA by the TCA cycle, promoting astaxanthin biosynthesis.

The TCA cycle is associated with the production of reactive oxygen species (ROS), which can stimulate the massive accumulation of astaxanthin in *X. dendrorhous* (Du et al. 2019; Zhang et al. 2019). In this study, the TCA cycle was inhibited by the addition of Na-citrate, while the inhibitory effect was weakened with the depletion of Na-citrate. These results indicated that the addition of Na-citrate could regulate the metabolic flux from the TCA cycle to carotenoid biosynthesis and regulate the citric acid-pyruvate cycle, providing a large amount of substrate and energy for cell growth and astaxanthin accumulation, thereby generating numerous ROS to enhance astaxanthin synthesis.

### Effect of Na-citrate on amino acid metabolism in *X. dendrorhous*

The amino acid content affects protein synthesis, which is closely related to the growth and reproduction of yeast. The content of amino acids was significantly increased with Na-citrate treatment in addition to aspartic acid (Table 2**)**. The content of alanine and serine, which are derived from pyruvate, increased in the Na-citrate group at different time points (Table 3). The contents of L-5-oxoproline, leucine, L-phenylalanine, and valine derived from intermediates of the TCA cycle also increased in the Na-citrate group. The addition of Na-citrate resulted in a significant decrease in intracellular aspartic acid content. Aspartic acid was closely related to the TCA cycle and could be oxidatively deaminated to generate oxaloacetate because the addition of Na-citrate could regulate the TCA cycle, which was more active after 72 h. More aspartic acid thus needed to be converted into substances in the TCA cycle for supplementation, which reduced the content of aspartic acid.

At the same time, many amino acid metabolites were much higher in the Na-citrate group compared with the control group (Table 3). Of note, the protein expressed (Additional file 1: Fig. S3) by *X. dendrorhous* was higher in the control group than in the Na-citrate group, suggesting that the higher-level amino acids were not used to synthesize the specific protein but to respond to the stress. This finding was consistent with that of Zhang et al., who reported that *Mortierella alpina* could adjust the metabolic state to rapidly change the pathways and adapt to new environments (Zhang et al. 2017). Furthermore, the amino acids showed higher abundance in Na-citrate cultures, suggesting that protein synthesis was restricted in *X. dendrorhous*. Acetyl-CoA is a key intermediate used in both primary and secondary metabolic pathways. Astaxanthin and fatty acid biosynthesis need acetyl-CoA, ATP, and NADPH as substrates, and thus their entry into these pathways must be regulated. The availability of acetyl-CoA, ATP, and NADPH may be key factors for switching the carbon flux from TCA-respiratory to astaxanthin biosynthesis during the restriction of protein synthesis in *X. dendrorhous*, increasing the accumulation of astaxanthin.

### Effect of Na-citrate on fatty acid and sterol metabolism in *X. dendrorhous*

In *X. dendrorhous*, carotenoid biosynthesis is closely associated with fatty acid metabolism by sharing the same precursor, acetyl-CoA (Du et al. 2019; Zhang et al. 2019). Herein, the contents of seven fatty acids in the Na-citrate groups were higher than those in the control group (Tables 2 and 3). The increased biomass was also correlated with an increased synthesis of fatty acids (Fig. 1). These results demonstrated that an appropriate Na-citrate feeding strategy might be an effective way to enhance astaxanthin accumulation in *X. dendrorhous*. The content of oleic acid, linoleic acid, and linoelaidic acid (unsaturated fatty acids) showed a significant increase (1.6–16.9 folds) in Na-citrate treatment groups at different culture stages. The content of hexadecanoic acid increased by 38.2-fold at 72 h in the Na-citrate treatment group, suggesting that Na-citrate facilitated the accumulation of fatty acids in *X. dendrorhous* and an increase in fatty acids, especially the unsaturated fatty acids. Unsaturated fatty acids enhance the fluidity and permeability of cell membranes (Los et al. 2013). The fatty acids increased before 72 h and showed a decreasing trend after 72 h. In the Na-citrate treatment groups, the high biomass caused insufficient nutrients in the medium. It is thus necessary to use part of the fatty acids for energy supply. Fatty acids may be used as a carbon source to enrich the acetyl-CoA supply for carotenoid biosynthesis. In this study, fatty acids were induced by Na-citrate to increase the fluidity and permeability of the cell membrane, thus accelerating the uptake of the glucose and nitrogen substrate from the medium into the cell, thereby promoting metabolism.

Metabolomics analysis showed that the addition of Na-citrate significantly changed the content of sterols (Tables 2 and 3). The contents of ergosterol and ergosta-7, 22-dien-3-ol in cells under Na-citrate treatment were 1.3- and 3.7-fold those of cells in the control group at 48 h. However, the contents of ergosterol and ergosta-7, 22-dien-3-ol decreased after 72 h. Ergosta-7, 22-dien-3-ol, and ergosterol play an important role in ensuring the integrity of the cell membrane, the activity of membrane-bound enzymes, membrane fluidity, cell viability, and cellular substance transport (Veen & Lang 2004). High content of sterols during the early stages of fermentation can thus cause the cells to better absorb nutrients from the medium, providing conditions for cell growth and accumulation of metabolites. Ergosterol can also compete with astaxanthin because they have the same precursor, farnesyl pyrophosphate (FPP) (Misawa 2011). Carotenoid and fatty acid syntheses share several common features with sterol synthesis, including the substrates of acetyl-CoA, ATP, and NADPH. Therefore, the content of ergosterol decreased during the later stages of fermentation. The content of ergosterol can regulate the expression of 3-hydroxy-3-methylglutaryl-CoA synthase (HMGS) and 3-hydroxy-3-methylglutaryl-CoA reductase (HMGR) in the mevalonate pathway. A high content of ergosterol can inhibit the expression of HMGR and HMGS. The addition of Na-citrate during the later phase can thus lead to a significant downregulation of ergosterol, thereby releasing the inhibition of key rate-limiting enzymes in the mevalonate pathway and directing the FPP in the metabolic pathway towards astaxanthin synthesis.

### Effect of Na-citrate on ROS level

**Fig. 5:**
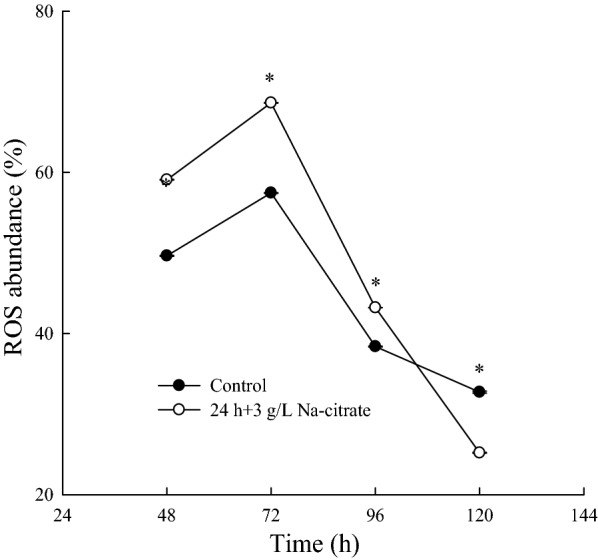
Intracellular reactive oxygen species (ROS) generation. The figure represents variation in ROS abundance with time, where * represents p < 0.05 compared to the control. The solid and hollow circles represent the intracellular ROS abundance in the control group and the Na-citrate group, respectively

Astaxanthin is a scavenger of free radicals, a chain-breaking antioxidant, and a potent quencher of ROS, such as singlet oxygen, superoxide ion, and hydrogen peroxide (Alesci et al. 2015; Martinez-Cardenas et al. 2018). The presence of astaxanthin means a higher survival ability of the cells because it enhances the resistance of the cell to oxidative stress. Astaxanthin biosynthesis thus serves as a survival strategy for *X. dendrorhous* under oxidative stress (Cuellar-Bermudez et al. 2015; Gessler et al. 2007). The ROS levels of both the control and Na-citrate groups increased, peaked at 72 h, and then decreased to a basal level at 120 h (Fig. 5). The production of ROS gradually increased with the enhanced metabolic activity of the yeast cells. Subsequently, the level of intracellular ROS gradually reduced with the production of astaxanthin, which can scavenge ROS. The ROS level was higher from 48 to 96 h in the Na-citrate group than in the control group (Fig. 5), suggesting that Na-citrate induced ROS accumulation, increased redox signaling, and induced synthesis of astaxanthin in *X. dendrorhous*.

In addition, the content of myo-inositol, a carbohydrate metabolism intermediate, in response to environmental stress was significantly upregulated before 72 h (Tables 2 and 3). Myo-inositol is a growth factor for yeast and contributes to responses to environmental factors, such as oxygen and osmotic pressure in *Aurantiochytrium* sp. and *Schizochytrium* sp. strains (Jakobsen et al. 2007; Yu et al. 2016a, b). In this study, Na-citrate treatment caused a significant increase in myo-inositol (2.1–3.9 folds) before 72 h, which was potentially a vital stress indicator in response to Na-citrate treatment. Further investigations are thus needed to determine the relationship between Na-citrate treatment and myo-inositol metabolism.

### Transcriptional responses of genes involved in astaxanthin biosynthesis

**Fig. 6:**
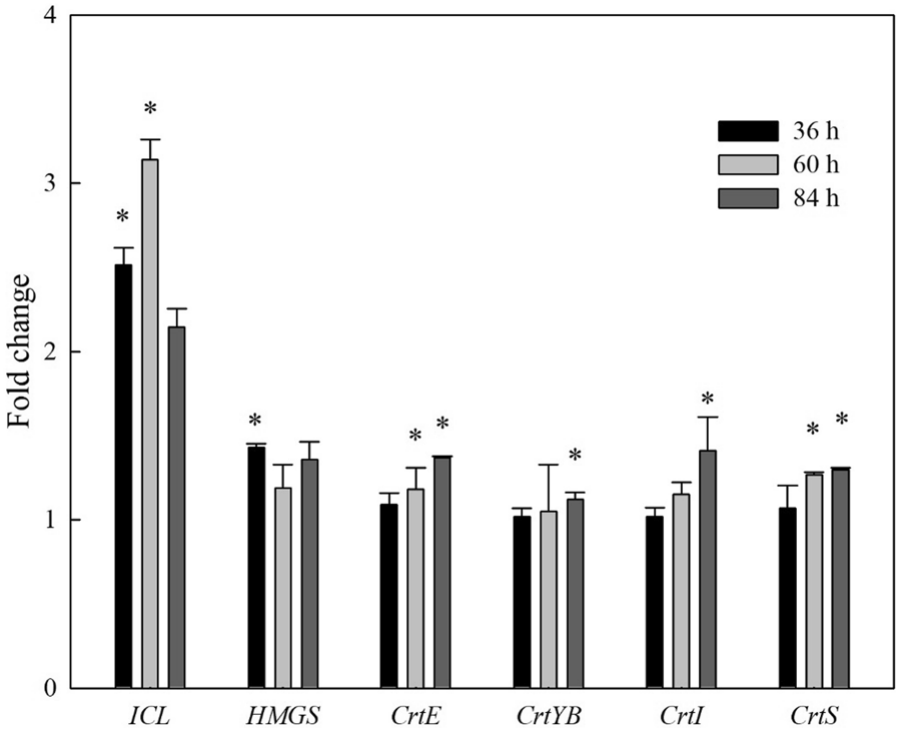
Na-citrate regulates the transcriptional level of key genes involved in astaxanthin synthesis. The key genes include *ICL* (encoding isocitrate lyase), *HMGS* (encoding HMG-CoA synthase), *crtE* (encoding GGPP synthase), *crtYB* (encoding phytoene synthase/lycopene cyclase), *crtI* (encoding phytoene dehydrogenase) and *crtS* (encoding astaxanthin synthase), where * represents statistical differences with P < 0.05 compared to the control. Values are means ± standard deviation of three independent experiments

The real-time PCR assay was used to detect the gene expression level of the astaxanthin biosynthesis pathway to explore the molecular mechanisms underlying the higher astaxanthin accumulation induced by Na-citrate. Six key genes, including *ICL* (encoding isocitrate lyase), *HMGS* (encoding HMG-CoA synthase), *crtE* (encoding GGPP synthase), *crtYB* (encoding phytoene synthase/lycopene cyclase), *crtI* (encoding phytoene dehydrogenase), and *crtS* (encoding astaxanthin synthase) were analyzed. The transcription of these genes was elevated by Na-citrate during the cultivation period (Fig. 6). ICL is a key enzyme in the glyoxylate cycle that splits isocitrate into glyoxylate and succinate. Glyoxylate combines with acetyl-CoA molecules to form malate. Compared to the control group, transcription of *ICL* in the Na-citrate group was increased at 36 h (2.51-fold), 60 h (3.14-fold), and 84 h (2.15-fold), indicating that the cells could convert substances, such as fatty acids into carbohydrates, thus promoting cell growth and product accumulation. The increased *HMGS* transcription under Na-citrate treatment suggested that Na-citrate treatment elevated the mevalonate pathway. The enhanced transcript level of *crtE*, *crtYB*, *crtI*, and *crtS* encoding the key enzymes for controlling the biosynthesis of astaxanthin under Na-citrate treatment during the entire cultivation period indicated that Na-citrate strengthened astaxanthin biosynthesis in *X. dendrorhous*.

### Metabolic mechanism of astaxanthin biosynthesis in *X. dendrorhous* in response to Na-citrate treatment

**Fig. 7:**
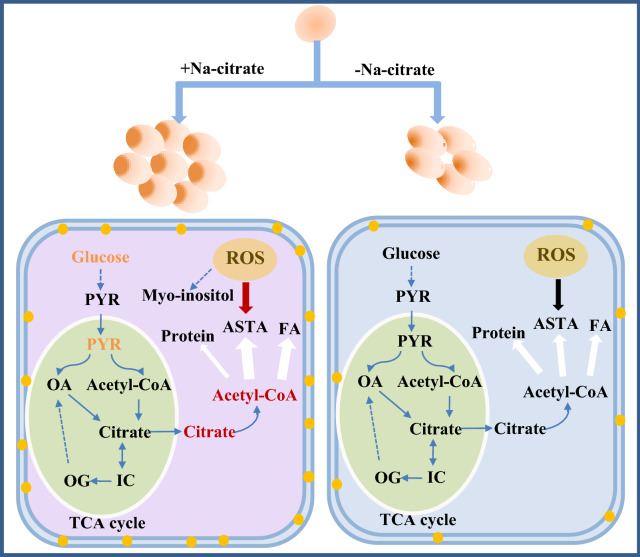
The mechanisms through which Na-citrate addition affects the cells of *X. dendrorhous*. PYR: pyruvate; OA: oxaloacetate; IC: isocitrate; OG: 2-oxo-glutarate; Asta: astaxanthin; FA: fatty acids

Increased biomass and astaxanthin accumulation were observed in *X. dendrorhous* under Na-citrate treatment. A comparison of the metabolites under the Na-citrate and control groups revealed that the metabolites content involved in the glycolysis pathway, amino acid metabolism, TCA cycle, and lipid and sterol biosynthesis changed substantially in response to Na-citrate. Figure 7 shows the metabolic mechanism of Na-citrate in regulating cell growth and astaxanthin accumulation.

Na-citrate treatment can promote the assimilation of glucose from the medium by cells. During the cultivation period, the consumption rate of intracellular glucose in the Na-citrate group was higher than that of the control group, indicating that the glycolysis flux was induced by Na-citrate so that intracellular glucose could generate more pyruvate. The increased glycolytic flux suggested that more glucose went through the pentose phosphate pathway (PPP pathway) to supply the increased demand of NADPH required for lipid synthesis and ROS increase. In addition, the flux of pyruvate to ethanol and lactic acid through anaerobic fermentation was weakened, allowing more pyruvate to be converted to acetyl-CoA for astaxanthin synthesis. Acetyl-CoA has several metabolic pathways. It can participate in the TCA cycle, astaxanthin, fatty acid, protein, and sterol syntheses. In this study, Na-citrate treatment increased the content of intracellular citric acid, thereby increasing the concentration of citric acid in the TCA cycle, which inhibited the catalytic activity of citrate synthase and weakened the reaction rate of oxaloacetate synthesis of citric acid. In contrast, the remaining Na-citrate in the mitochondria entered the cytoplasm and was cleaved into acetyl-CoA. The significant increase in *ICL* transcription also suggested that the content of acetyl-CoA in the cytoplasm was increased, thereby providing numerous substrates for the production of astaxanthin in *X. dendrorhous*.

Na-citrate treatment significantly increased intracellular ROS. The accumulated astaxanthin increased the resistance of *X. dendrorhous* to Na-citrate stress by removing ROS species because of its strong antioxidant activity, which increased redox signaling and induced astaxanthin synthesis in *X. dendrorhous*. Furthermore, Na-citrate treatment significantly upregulated the expression of the other five key genes involved in carotenogenesis. Astaxanthin is synthesized in *X. dendrorhous* via the mevalonate pathway, in which HMGS is a rate-limiting enzyme that catalyzes the formation of HMG-CoA. In this study, the significant increase in HMGS transcription suggested that the mevalonate pathway was increased, which was consistent with the enhancement of astaxanthin accumulation in *X. dendrorhous*.

The regulatory mechanism proposed that Na-citrate treatment increases the use of glucose for the fermentation based on the biochemical compositions and metabolome analysis, indicating that Na-citrate induced a glycolysis flux. Upregulation of the glycolytic pathway suggested that more glucose went through the PPP pathway to improve the NADPH for astaxanthin biosynthesis. Notably, Na-citrate treatment increased the content of intracellular citric acid but reduced the metabolites in the TCA cycle. Exogenous Na-citrate may provide more acetyl-CoA by cleaving citrate to produce acetyl-CoA, thus reducing the consumption of acetyl-CoA via the TCA cycle, thereby promoting astaxanthin and fatty acids biosynthesis in *X. dendrorhous*. Na-citrate treatment significantly increased intracellular ROS, which increased redox signaling and further induced astaxanthin accumulation in *X. dendrorhous*. Additionally, the upregulation of the six genes encoding key enzymes involved in astaxanthin biosynthesis was potentially caused by the increase in their substrates and higher levels of ROS because of Na-citrate treatment.

## Conclusions

*Xanthophyllomyces dendrorhous* can produce large amounts of astaxanthin, which is a high-value ketocarotenoid. This study revealed that Na-citrate treatment could promote astaxanthin production in *X. dendrorhous* with a twofold increase. Metabolic Analysis revealed that Na-citrate treatment increased the use of glucose for fermentation and weakened the intracellular TCA cycle, thus promoting the metabolic flux from acetyl-CoA to astaxanthin biosynthesis. This finding was consistent with the increased transcriptional expression of six key genes (*ICL*, *HMGS*, *crtE*, *crtYB*, *crtI,* and *crtS*) associated with carotenoid biosynthesis pathways. The increased ROS abundance also indicated that Na-citrate treatment potentially induced the anti-stress mechanism in *X. dendrorhous* to produce more astaxanthin. These results provide a potentially valuable strategy for stimulating astaxanthin production in *X. dendrorhous* using exogenous Na-citrate. A fed-batch feeding employing the Na-citrate strategy for astaxanthin production in *X. dendrorhous* should thus be considered in future studies.

### Supplementary Information

**Additional file 1: Figure S1.** Effect of 2 g/L Na-citrate addition at different times on the growth and astaxanthin production of *X. dendrorhouos*. (A) Biomass (g/L); (B) Carotenoids titer (mg/L); (C) Astaxanthin titer (mg/L); (D) Astaxanthin content (mg/g). The cells were grown in a 250-mL Erlenmeyer flask containing 50 mL fermentation medium, with the temperature maintained at 22°C and the stirring speed at 200 rpm. Values are mean ± standard deviation of three independent experiments. **Figure S2.** Effect of different Na-citrate concentrations at 24 h on the growth and astaxanthin production of *X. dendrorhouos*. (A) Biomass (g/L); (B) Carotenoids titer (mg/L); (C) Astaxanthin titer (mg/L); (D) Astaxanthin content (mg/g). The cells were grown in a 250-mL Erlenmeyer flask containing 50 mL fermentation medium, with the temperature maintained at 22°C and the stirring speed at 200 rpm. Values are mean ± standard deviation of three independent experiments. **Figure S3.** Na-citrate regulates the protein content in *X. dendrorhouos*. fold change is the ratio of the protein content of the control group to the Na-citrate group. **Table S1.** Gene-specific primers used for RT-qPCR; F: Forward; R: Reverse

## Data Availability

Data generated and analyzed in this study are included in the published article and the supplementary materials.
