# Supplementary material for: Metabolic mechanism of astaxanthin biosynthesis in Xanthophyllomyces dendrorhous in response to sodium citrate treatment
Source: Bioresour Bioprocess. 2023 Apr 26;10(1):29. doi: 10.1186/s40643-023-00650-7 (PMC10992204; doi:10.1186/s40643-023-00650-7)
Supplement: Supplementary file 1 — Additional file 1: Figure S1. Effect of 2 g/L Na-citrate addition at different times on the growth and astaxanthin production of X. dendrorhouos. (A) Biomass (g/L); (B) Carotenoids titer (mg/L); (C) Astaxanthin titer (mg/L); (D) Astaxanthin content (mg/g). The cells were grown in a 250-mL Erlenmeyer flask containing 50 mL fermentation medium, with the temperature maintained at 22°C and the stirring speed at 200 rpm. Values are mean ± standard deviation of three independent experiments. Figure S2. Effect of different Na-citrate concentrations at 24 h on the growth and astaxanthin production of X. dendrorhouos. (A) Biomass (g/L); (B) Carotenoids titer (mg/L); (C) Astaxanthin titer (mg/L); (D) Astaxanthin content (mg/g). The cells were grown in a 250-mL Erlenmeyer flask containing 50 mL fermentation medium, with the temperature maintained at 22°C and the stirring speed at 200 rpm. Values are mean ± standard deviation of three independent experiments. Figure S3. Na-citrate regulates the protein content in X. dendrorhouos. fold change is the ratio of the protein content of the control group to the Na-citrate group. Table S1. Gene-specific primers used for RT-qPCR; F: Forward; R: Reverse [file 40643_2023_650_MOESM1_ESM.docx]

**Figure. S1** Effect of 2 g/L Na-citrate addition at different times on the growth and astaxanthin production of *X. dendrorhouos*. (A) Biomass (g/L); (B) Carotenoids titer (mg/L); (C) Astaxanthin titer (mg/L); (D) Astaxanthin content (mg/g). The cells were grown in a 250 mL Erlenmeyer flask containing 50 mL fermentation medium, with the temperature maintained at 22°C and the stirring speed at 200 rpm. Values are mean ± standard deviation of three independent experiments.

**Figure. S2** Effect of different Na-citrate concentrations at 24 h on the growth and astaxanthin production of *X. dendrorhouos*. (A) Biomass (g/L); (B) Carotenoids titer (mg/L); (C) Astaxanthin titer (mg/L); (D) Astaxanthin content (mg/g). The cells were grown in a 250 mL Erlenmeyer flask containing 50 mL fermentation medium, with the temperature maintained at 22°C and the stirring speed at 200 rpm. Values are mean ± standard deviation of three independent experiments.

**Figure. S3** Na-citrate regulates the protein content in *X. dendrorhouos*. fold change is the ratio of the protein content of the control group to the Na-citrate group.

**Table S1** Gene-specific primers used for RT-qPCR; F: Forward; R: Reverse.

| **Gene Name** | **Primers: F** | **Primers: R** |
| --- | --- | --- |
| *ICL*(Isocitrate lyase) | 5’-CCTCATCATCTTCCCTATTGCA-3’ | 5’-CAATTCCGAACGCAACCGGCT-3’ |
| *HMGS* (HMG-CoA synthase) | 5’-CGCCTTGAACGCTGTTTCCGG-3’ | 5’-ACCCTCGGCGTAGATGGCAAT-3’ |
| *crtE* (GGPP synthase) | 5’-GATTACGCGAACATCCTCACAGCAA-3’ | 5’-CAACAACGTTCTGGATGACCTCGAG-3’ |
| *crtYB* (Phytoene synthase) | 5’-ACGGCTCTCGCATATTACCAGATCC-3’ | 5’-CGCCATTTCTGATGATCCATGAGTC-3’ |
| *crtI* (Phytoene dehydrogenase) | 5’-CACAGCTATCATCGTGGGATGTGG-3’ | 5’-ATCTGGCAAGAGCAGCAAACTGGG-3’ |
| *crtS* (Astaxanthin synthase) | 5’-TCATCTTGGTCTTGCTCACAGGTGC-3’ | 5’-TCGCATGCTCTTCACCTGTACGAG-3’ |
| *act* (Actin) | 5’-CCGCCCTCGTGATTGATAAC-3’ | 5’-TCACCAACGTAGGAGTCCTT-3’ |
